# The Antibacterial and Anti-Eukaryotic Type VI Secretion System MIX-Effector Repertoire in *Vibrionaceae*

**DOI:** 10.3390/md16110433

**Published:** 2018-11-04

**Authors:** Yasmin Dar, Dor Salomon, Eran Bosis

**Affiliations:** 1 Department of Clinical Microbiology and Immunology, Sackler Faculty of Medicine, Tel Aviv University, Tel Aviv 6997801, Israel; yasmindar@mail.tau.ac.il; 2 Department of Biotechnology Engineering, ORT Braude College of Engineering, Karmiel 2161002, Israel

**Keywords:** T6SS, vibrio, MIX-effector, toxin, secretion, virulence, antibacterial

## Abstract

*Vibrionaceae* is a widespread family of aquatic bacteria that includes emerging pathogens and symbionts. Many *Vibrionaceae* harbor a type VI secretion system (T6SS), which is a secretion apparatus used to deliver toxins, termed effectors, into neighboring cells. T6SSs mediate both antibacterial and anti-eukaryotic activities. Notably, antibacterial effectors are encoded together with a gene that encodes a cognate immunity protein so as to antagonize the toxicity of the effector. The MIX (Marker for type sIX effectors) domain has been previously defined as a marker of T6SS effectors carrying polymorphic C-terminal toxins. Here, we set out to identify the *Vibrionaceae* MIX-effector repertoire and to analyze the various toxin domains they carry. We used a computational approach to search for the MIX-effectors in the *Vibrionaceae* genomes, and grouped them into clusters based on the C-terminal toxin domains. We classified MIX-effectors as either antibacterial or anti-eukaryotic, based on the presence or absence of adjacent putative immunity genes, respectively. Antibacterial MIX-effectors carrying pore-forming, phospholipase, nuclease, peptidoglycan hydrolase, and protease activities were found. Furthermore, we uncovered novel virulence MIX-effectors. These are encoded by “professional MIXologist” strains that employ a cocktail of antibacterial and anti-eukaryotic MIX-effectors. Our findings suggest that certain *Vibrionaceae* adapted their antibacterial T6SS to mediate interactions with eukaryotic hosts or predators.

## 1. Introduction

*Vibrionaceae* is a widespread family of aquatic Gram-negative bacteria, to which the genera *Vibrio*, *Aliivibrio*, *Photobacterium*, and others belong [1]. Many members of this family are emerging human pathogens (e.g., *V. cholerae*, *V. parahaemolyticus*, and *V. vulnificus*) and marine animal pathogens (e.g., *V. anguillarum* and *V. coralliilyticus*) [2,3,4], whereas others are marine animal symbionts (e.g., *Aliivibrio fischeri*) [5]. An increase in *Vibrionaceae* abundance and in the number of disease incidence caused by these pathogens was observed in the past half-century [6]. Interestingly, this increase was linked to the world-wide rise in ocean water temperature, implying that a further rise in water temperature may intensify the spread of *Vibrionaceae* and disease occurrence [6]. Importantly, members of this family were shown to cause disease not only as individual clones, but also as consortia [7].

*Vibrionaceae* carry diverse arsenals of virulence factors, such as adhesins, secreted toxins, type III secretion systems (T3SS), and type VI secretion systems (T6SS) [8,9]. T6SS is a protein delivery machinery that is widely distributed among Gram-negative bacteria [10,11,12]. T6SSs deliver toxins, termed effectors, directly into neighboring cells [13]. Effectors can mediate both the antibacterial activities and anti-eukaryotic activities, thus implicating T6SSs in bacterial competition and host-pathogen interactions, respectively [14,15,16]. Whereas T6SS was originally characterized as a virulence mechanism in *V. cholerae* [12] and *Pseudomonas aureginosa* [11], the current consensus is that most T6SSs mediate antibacterial activities [17]. Bacteria protect themselves against effector-mediated self-intoxication by using adjacently encoded immunity proteins that bind to their cognate antibacterial effectors and antagonize their activity [15,18].

The role of T6SSs in antibacterial competition and virulence has been characterized in several *Vibrionaceae* species, among them *V. cholerae* [12,19], *V. parahaemolyticus* [20], *V. alginolyticus* [21], *V. proteolyticus* [16], *V. fluvialis* [22], *V. anguillarum* [23], *Aliivibrio fischeri* [24], and *V. vulnificus* [25]. All *Vibrionaceae* T6SSs that have been studied to date exhibit antibacterial activities by delivering effectors carrying various catalytic domains, such as nucleases [26], peptidoglycan hydrolyses [27,28], phospholipases [21], and pore-forming toxin domains [29]. T6SSs in at least two *Vibrio* species, *V. cholerae* and *V. proteolyticus*, also deliver effectors that mediate activities against eukaryotic cells [14,16,30]. Thus, it is plausible that other *Vibrionaceae* also utilize their T6SSs against both bacteria and eukaryotes.

We previously described a polymorphic class of T6SS effectors, termed MIX-effectors. MIX-effectors harbor an N-terminal domain, termed MIX (Marker for type sIX effectors), fused to polymorphic C-terminal toxin domains [26]. MIX-domains can be divided into five clans (termed MIX I–V) [26]. Members of the MIX V clan are shared between marine bacteria via horizontal gene transfer, thereby enhancing their bacterial competitive fitness [21]. Whereas most MIX-effectors identified to date are predicted to mediate antibacterial toxicity [16,21,26], we recently found that a member of the MIX V clan that is encoded by *V. proteolyticus*, VPR01S_11_01570, carries a CNF1 (cytotoxic necrotizing factor 1) toxin domain and modulates the actin cytoskeleton of eukaryotic phagocytic cells [16]. In addition, VasX, a clan IV MIX-effector that is encoded by *V. cholerae*, was originally identified as being required for toxicity against grazing amoeba [30]. Taken together, these findings suggest that bacteria employ polymorphic MIX-effectors to target eukaryotes, potentially via yet unknown toxin domains and mechanisms.

The repertoire of T6SS effectors differs between isolates of the same species [21,24,31,32]. As hundreds of new *Vibrionaceae* genome sequences have become available since the discovery of MIX in 2014 [26], we hypothesized that yet unknown MIX-effectors are found in the *Vibrionaceae* pan-genome. Here, we set out to characterize the pan-*Vibrionaceae* MIX-effector repertoire, searching for novel effectors and focusing on those that may target eukaryotes. Using a computational approach, we searched all publicly available *Vibrionaceae* genomes, and identified those genes encoding MIX-effectors. We describe various MIX-effector families with both predicted antibacterial activities and anti-eukaryotic toxin domains. We coined the term “professional MIXologists” to describe bacterial strains that encode numerous MIX-effectors (as they employ a cocktail of MIX-effectors). Based on our findings, we propose that certain *Vibrionaceae* “professional MIXologists” adapted their T6SSs to mediate not only antibacterial activities, but also interactions with their eukaryotic hosts or as a defense against eukaryotic predators.

## 2. Results and Discussion

### 2.1. Identifying MIX-Effectors in Vibrionaceae

**Figure 1:**
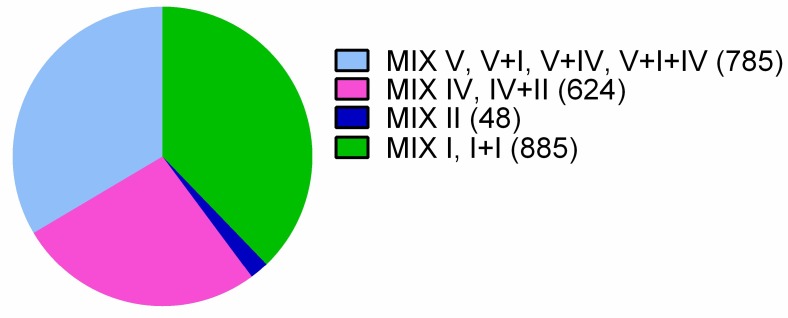
Pie chart representing a clan association of identified *Vibrionaceae* MIX-effectors. The number of proteins associated with each clan is shown in parentheses.

The RefSeq database includes 2994 sequenced *Vibrionaceae* genomes that have been assembled to various degrees (Dataset S1). We employed reverse position-specific BLAST (RPS-BLAST) [33] to identify the MIX-containing proteins in *Vibrionaceae* genomes. In total, we identified 2342 MIX-containing proteins encoded by 1311 *Vibrionaceae* genomes (Dataset S2). For each MIX domain that was identified, we determined the clan to which it belonged (MIX I–V) [26], as well as its position within the protein sequence. Of the 2342 MIX-effectors identified, 848 contained MIX I, 48 contained MIX II, 623 contained MIX IV, and 597 contained MIX V. We also identified 226 proteins that contained multiple MIX domains (Figure 1, Dataset S2). We did not detect any MIX III clan members in *Vibrionaceae*.

### 2.2. *Vibrionaceae* MIX-Effectors Carry Diverse C-Terminal Toxin Domains

MIX-effectors contain polymorphic C-terminal toxin domains [26]. In order to identify and characterize the toxin domains that are carried by MIX-effectors, we clustered the C-terminal domains of the MIX-effectors, using CD-HIT [34], based on the sequences located C-terminally to the MIX domains (Dataset S2 and S3). The C-terminal sequences of the MIX-effectors can be grouped into 86 distinct clusters. We reasoned that the MIX-effectors that mediate the antibacterial activities are part of bicistronic units that also encode cognate immunity proteins to antagonize the effectors [15,18]. The cognate immunity genes of MIX-effectors that have been described to date are encoded downstream of the effector [21,26], possibly reflecting the C-terminal position of the toxin domain within the effector. Therefore, we premised that the cognate immunities would be encoded by the genes downstream of the MIX-effectors on the same strand. We identified all of the instances in which genes are located downstream of the MIX-effector on the same strand (Dataset S2, S3). Moreover, we made no assumption with regard to the distance of the downstream genes from the MIX-effector, because in many genes, the translation start site was mis-annotated. Most MIX-effectors belong to clusters in which the MIX-effectors were part of bicistronic units, suggesting that they are antibacterial toxins and that their downstream-encoded proteins could serve as their cognate immunities. Interestingly, we identified MIX-effector clusters that did not have predicted immunity genes downstream. They were suspected to carry anti-eukaryotic toxin domains (discussed next).

With the above observations in mind, we analyzed the MIX-effector clusters and identified putative activities for their C-terminal toxin domains. To this end, we employed both automated annotation, using the NCBI conserved domain database (CDD) [35], and a manual examination of cluster representatives, using HHpred remote homology detection and a structure prediction server provided by the MPI Bioinformatics Toolkit [36]. The toxin domains that we identified are summarized in Table 1 and in Dataset S3. The largest group, with representatives from all MIX clans, consists of MIX-effectors containing transmembrane helices at their C-termini, which are predicted to be pore-forming toxins similar to the antibacterial pore-forming bacteriocins [37]. The second largest group consists of MIX I members that are similar, in both sequence and synteny, to the *V. parahaemolyticus* MIX-effector VP1388 [26]; their activity remains unknown. Many MIX-effectors that belong to the MIX V clan harbor C-terminal nuclease domains, whereas others have domains with predicted phospholipase, peptidoglycan-hydrolase, or peptidase activities. We also identified MIX I effectors with a C-terminal nucleotide deaminase domain. We were unable to assign potential toxin activities to several MIX-effectors, even though they are predicted to mediate antibacterial toxicity as they are encoded in bicistronic units. We observed that the effectors predicted to exert their toxicity on the cell membrane or in the periplasm (i.e., pore-forming, phospholipases, and peptidoglycan-hydrolases) were accompanied by downstream immunity proteins predicted to localize to these cell compartments (i.e., they contain transmembrane helices or an N-terminal signal peptide) (Dataset S2). This observation supports the predicted activities of the toxin domains.

We also identified 124 MIX-containing proteins that appear to have no C-terminal toxin domain. A large group of these proteins share sequence similarity with MIX-effectors that have DUF2235 (predicted phospholipase [38]) C-terminal domains, but they appear to be truncated right before the beginning of the toxin domain. Others are either truncated with a downstream open reading frame encoding a toxin domain (which could be actual distinct open reading frames or result from sequencing or assembly errors), followed by a cognate immunity gene, or they simply do not possess a C-terminal toxin extension (referred to hereafter as truncated). Many of the latter ones have no downstream open reading frame that could encode a cognate immunity, but as no toxin domain is present, we did not consider them as putative anti-eukaryotic effectors. All together, our results support the notion that has been previously put forward [26], that MIX-effectors are polymorphic modular proteins composed of MIX domains and interchangeable C-terminal toxin domains. In light of this, it remains to be determined whether the truncated MIX-containing proteins are functional and play a role in T6SS activities. We speculate that they may simply be intermediate forms awaiting an interchangeable C-terminal toxin module to be fused to them. Another possibility is that these truncated forms are degenerated MIX-effectors that are no longer functional and have lost their toxin domain.

We were left with 27 MIX-effectors, belonging to the MIX V and MIX I clans, that contain a C-terminal extension, but no adjacent putative immunity gene, neither upstream nor downstream. We hypothesized that these 27 MIX-effectors target eukaryotic cells. Indeed, one of them is *V. proteolyticus* VPR01S_11_01570, a MIX-effector that was shown to target eukaryotic phagocytic cells [16]. The analysis of these predicted virulence MIX-effectors is further discussed in the following section.

**Figure 2:**
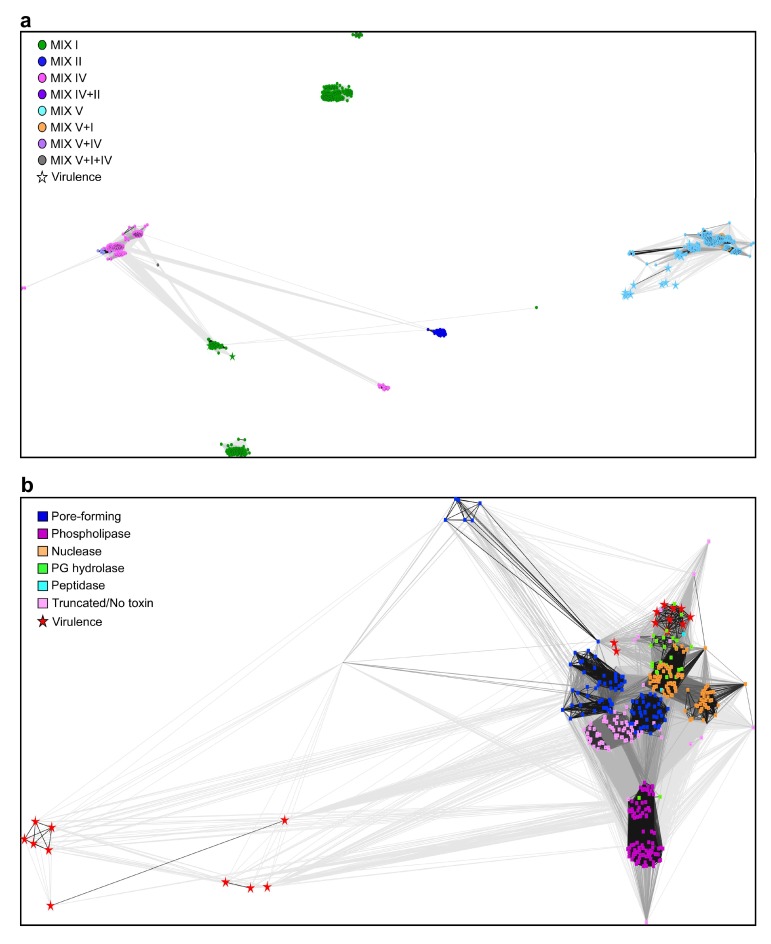
Grouping of *Vibrionaceae* MIX-effector C-termini using CLANS. (**a**) C-termini of MIX-effectors identified in *Vibrionaceae* clustered in three dimensions and colored according to the clans of their N-terminal-fused MIX domains; (**b**) C-termini of MIX-effectors containing MIX V or MIX V and I domains clustered in two dimensions and colored according to their predicted activity. Clustering was performed based on all-against-all sequence similarity, with nodes representing each sequence and connecting lines representing the distances between sequences. PG = peptidoglycan.

Next, we set out to better understand the connections between the different MIX clans and the C-terminal toxin domains. To this end, we grouped the C-termini of the MIX-effectors using CLANS [39], and colored each protein according to the clan of its MIX domain (Figure 2a). Surprisingly, we found that the C-termini were grouped according to the MIX clans to which they were fused. Most C-termini of the MIX-effectors that contain a MIX IV (or MIX V and IV) domain were grouped close together, and were connected to those containing a MIX II domain and to a group that contains a MIX I domain. The vast majority of the proteins in these groups are predicted to have pore-forming toxins with C-terminal transmembrane helices. Three additional distinct groups containing MIX I domains are also apparent. The two larger ones are largely composed of homologs of the *V. parahaemolyticus* MIX-effector, VP1388 [26]. The C-termini of the MIX V clan members were grouped together more loosely, and were distinct from the other groups (Figure 2a). The grouping of the MIX-effector C-termini according to the identity of the MIX clan could have resulted from two scenarios, as follows: (i) additional intermediate sequences between MIX and the toxin play a role in the classification of MIX-effectors; (ii) certain toxin domains are preferentially fused to specific clans of MIX domains. We think that a combination of both possibilities exists. Whereas we observed specific activities linked to MIX I, II, and IV clans, a close examination of the group containing the MIX V clan members revealed that they are sub-grouped, mostly based on the predicted activity of the C-terminal toxin domain (Figure 2b). Therefore, we can conclude that both phenomena occur. Whereas certain MIX clans prefer specific toxin domains and activities, MIX V is more “promiscuous” and can be plugged into a large variety of toxin domains and activities, including predicted anti-eukaryotic toxin domains.

### 2.3. Predicted Anti-Eukaryotic MIX-Effector Families

Of the predicted virulence MIX-effectors mentioned above, 25 belong to the MIX V clan and two belong to the MIX I clan (Table 1 and Dataset S3). The 27 putative virulence MIX-effectors are found in eight CD-HIT-generated clusters (Table 2). One effector, which constitutes cluster 83, is the previously reported *V. proteolyticus* CNF1-containing, virulence MIX-effector VPR01S_11_01570 (WP_040902815.1). It is a MIX V effector that targets phagocytic eukaryotic cells [16]. Its presence in the list of predicted virulence MIX-effectors validates our approach.

Within the remaining 26 predicted virulence effectors, 10 effectors belonging to cluster 27 (e.g., WP_065611703.1) are found in *Aliivibrio* species (*wodanis*, *logei*, and *sifiae*). They contain a MIX V domain, followed by LysM and PG-binding_1 domains (predicted to bind peptidoglycans), as well as a C-terminal domain similar to the protease domain of Anthrax lethal toxin [40,41] (Figure S1a). These toxins are encoded upstream of a T6SS gene cluster similar to *V. parahaemolyticus* T6SS1 [20]. Three effectors belonging to cluster 55 (e.g., WP_005429108.1) are found in *V. campbellii*. They contain a MIX V domain and a C-terminal protease domain similar to the cysteine protease domains of the *V. cholerae* RtxA [42] and the *Clostridium difficile* TcdB [43] virulence toxins, which are activated inside a host cell upon binding the eukaryotic-specific co-factor inositol hexakisphosphate (InsP_6_) [43] (Figure S1b). Two effectors belonging to cluster 53 (e.g., WP_012535377.1) are found in *A. fischeri*. They contain a MIX V domain, followed by LysM domains, and a C-terminal domain that is predicted to structurally resemble the peptidase M23 domain; however, the catalytic residues are not conserved [44] (Figure S1c). Interestingly, the protein encoded upstream of these MIX-effectors is a virulence toxin that contains a C-terminal Tox-PLDMTX superfamily domain (dermonecrotoxin of the papain-like fold) [45]. Five effectors belonging to cluster 38 (e.g., WP_006962196.1) are found in *V. coralliilyticus*. They contain a MIX V domain and a C-terminal domain that bears a weak similarity to glycosyltranferases, although the catalytic residues do not appear to be conserved. Remarkably, this C-terminal part is homologous to an unannotated region within the *Chromobacterium amazonense* MARTX virulence toxin RtxA (WP_083340985.1), and to a *Chromobacterium violaceum* protein (SUX55948.1) that is encoded within a virulence T3SS gene cluster (Figure S1d). Four effectors belonging to cluster 58 (e.g., WP_021709833.1) are found in *V. proteolyticus* and *V. azeurus*. They contain a MIX V domain and a C-terminal domain of an unknown function, which is homologous to a previously described insecticidal virulence toxin of *Photorhabdus*, Plu1690 [46] (Figure S1e). In *V. proteolyticus*, this MIX-effector is encoded adjacent to the CNF1-containing virulence MIX-effector VPR01S_11_01570 [16]. These *V. corallilyticus* MIX-effectors are encoded within the previously defined Coralliilyticus pathogenicity island-1 (CPI-1), which contains other virulence toxins such as cytolysin, RTX toxin, and T3SS gene clusters [47]. Two additional putative virulence MIX-effectors, encoded by *V. aerogenes*, and constituting clusters 72 (WP_073603189.1) and 76 (WP_073605246.1), contain a MIX I domain fused to C-terminal domains with transmembrane helices. We predict that they are pore-forming toxins that specifically target eukaryotic membranes. Taken together with the predicted C-terminal toxin domains that are similar to known virulence toxins, our findings of other virulence toxins adjacent to the predicted virulence MIX-effector, as well as the presence of some MIX-effectors within established pathogenicity islands, support our prediction of their anti-eukaryotic purpose.

**Figure 3:**
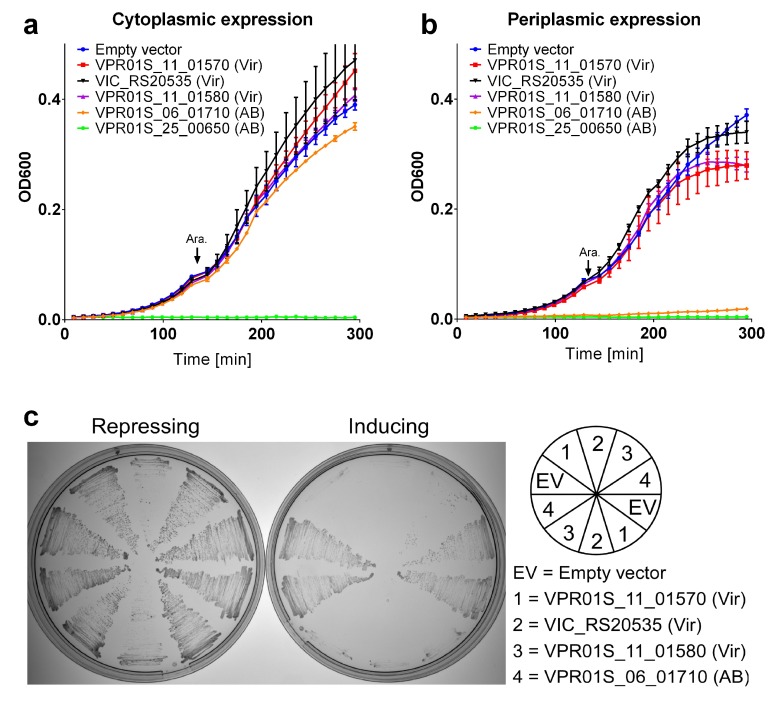
Toxicity of MIX-effectors in bacteria and yeast. (**a**,**b**) Growth of *E. coli* BL21 (DE3) containing arabinose-inducible vectors for expression of the indicated MIX-effectors with a C-terminal c-Myc/6xHis tag in the cytoplasm (**a**) or periplasm (**b**). Vir—predicted virulent. AB—predicted antibacterial. Ara denotes the time at which the inducer, L-arabinose, was added to the media. (**c**) *S. cerevisiae* strains harboring an empty vector or the indicated galactose-inducible MIX-effector expression plasmids were streaked onto selective media plates containing glucose (repressing) or galactose and raffinose (inducing).

Next, we set out to validate our virulence MIX-effector predictions. To this end, we selected two predicted antibacterial and two predicted anti-eukaryotic MIX-effectors, encoded by bacterial strains that were available in our laboratory, together with VPR01S_11_01570, which served as an anti-eukaryotic T6SS effector positive control [16]. The selected virulence MIX-effectors belong to clusters 38 (VIC_RS20535) and 58 (VPR01S_11_01580). The chosen antibacterial effectors are predicted to exert their toxic effect in the bacterial cytoplasm (VPR01S_25_00650 from cluster 4) or periplasm (VPR01S_06_01710 from cluster 82). First, we set out to demonstrate that the predicted virulence MIX-effectors do not mediate antibacterial activities. Indeed, their expression in bacteria, either in the cytoplasm or in the periplasm, was not detrimental. In contrast, the predicted antibacterial effectors were toxic when expressed in their designated target compartment (Figure 3a,b). Notably, VPR01S_25_00650, which was predicted to function in the bacterial cytoplasm, was toxic both in the cytoplasm and when it was fused to the PelB signal peptide that destined it to the periplasm (Figure 3b). We suspect that the apparent toxicity of the periplasmic version of VPR01S_25_00650 resulted from activity in the cytoplasm prior to its delivery to the periplasm. The yeast *Saccharamyces cerevisiae* has been extensively used as a surrogate eukaryotic model to study the virulence of bacterial toxins [48,49,50,51,52,53]. Therefore, we used yeast as a model eukaryotic cell so as to examine the virulence potential of the selected predicted anti-eukaryotic MIX-effectors. The expression of the two predicted virulence MIX-effectors inhibited yeast growth, as did the CNF1-containing VPR01S_11_01570 (Figure 3c). In contrast, the expression of the antibacterial MIX effector, VPR01S_06_01710, had no effect on yeast growth. Attempts to clone the antibacterial MIX-effector, VPR01S_25_00650, into a yeast expression vector were unsuccessful, possibly because of a leaky expression of the toxin in *E. coli*. Notably, the lack of toxicity did not result from a lack of MIX-effector expression, as evident from immunoblots (Figure S2). However, we did not detect the expression of effectors when their expression proved detrimental to either *E. coli* or yeast (Figure S2). Our inability to detect the expression of the detrimental effectors resulted from their inability to accumulate to detectable levels as the cells in which they were expressed did not grow. These results support our prediction of *Vibrionaceae* virulence MIX-effectors, and indicate that these toxins target a eukaryotic cell process that is conserved in yeast. Their exact target and use in the context of a host–bacterium interaction remains to be determined.

### 2.4. MIX-Effectors Are Unevenly Distributed Among Vibrionaceae Species

**Figure 4:**
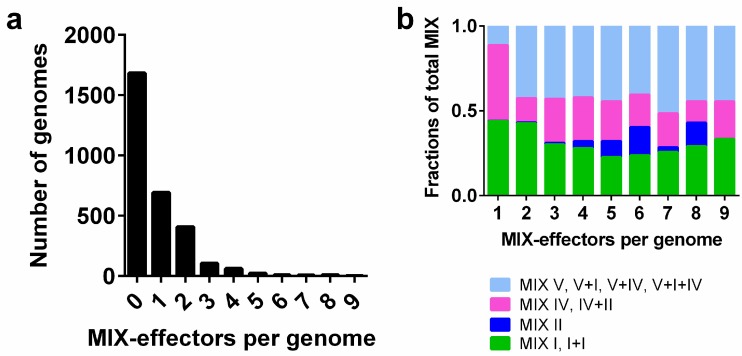
MIX-effectors’ distribution in *Vibrionacea* genomes. (**a**) Numbers of *Vibrionacea* genomes encoding the indicated number of MIX-effectors; (**b**) distribution of MIX clans within genomes with the indicated number of MIX-effectors.

Of the 2994 *Vibrionaceae* genomes that we analyzed, only 1311 genomes encode MIX-effectors. Most of the *Vibrionaceae* genomes that encode MIX-effectors have one or two MIX-effectors, and only a few genomes contain more than four MIX-effectors (Figure 4a). We observed that MIX V-effectors are more abundant in the genomes encoding two or more MIX-effectors (Figure 4b), implying their role in diversifying the MIX-effector repertoires in genomes that encode multiple MIX-effectors. However, the ratio of the different MIX clans was similar among genomes that encode multiple MIX-effectors, indicating that the effector repertoires are diversified by members of all MIX clans.

**Figure 5:**
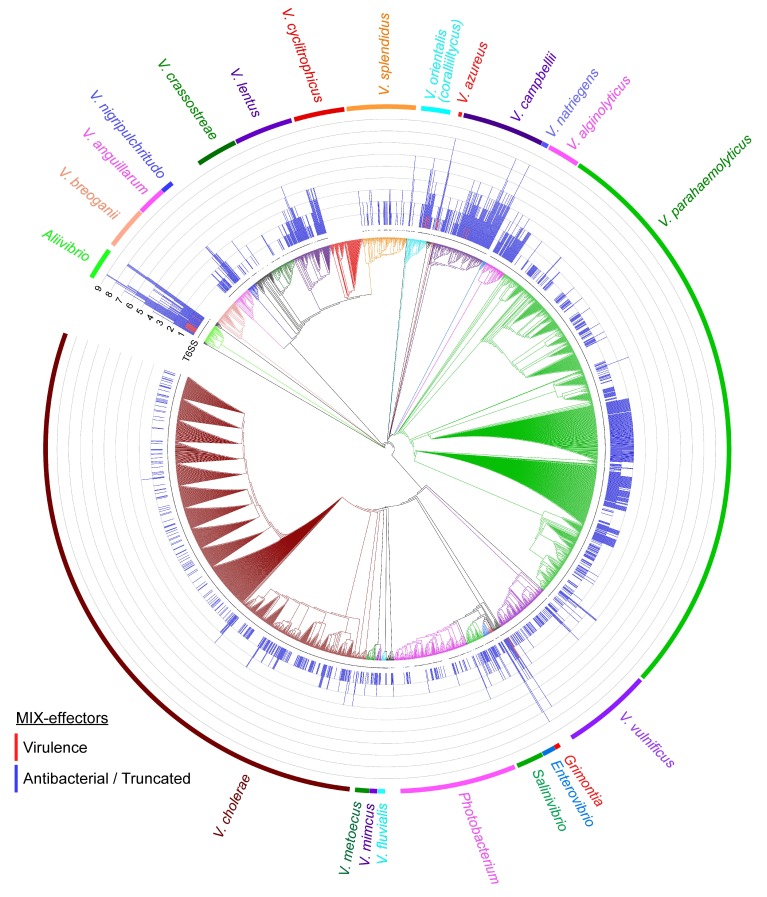
Distribution of MIX-effectors and T6SS in *Vibrionaceae*. The phylogenetic tree was based on the DNA sequences of *rpoB* coding for DNA-directed RNA polymerase subunit beta. The evolutionary history was inferred using the neighbor-joining method (see Materials and Methods for details). Blue bars indicate the number of MIX-effectors per genome (between 0 and 9). Red bars indicate the number of predicted virulence MIX-effectors. Black dots below bars indicate the presence of T6SS. Nodes containing species of interest are colored.

An analysis of the distribution of T6SS revealed that, in general, all of the genomes that encode MIX-effectors also encode T6SS (aside from isolated instances that could have resulted from low sequence coverage of the genomes) (Figure 5 and Dataset S4). This result supports previous observations [26] and strengthens the link between MIX-effectors and T6SS. Several species encode neither T6SS nor MIX-effectors (e.g., *V. breoganii*, *V. cyclitrophicus*, and *V. natriegens*). Some species, such as *V. cholerae*, *V. parahaemolyticus*, and *V. vulnificus*, employ MIX-effectors, but the distribution of MIX-effectors across the different isolates varies. Whereas some isolates encode one or two MIX-effectors, other isolates have no MIX-effectors. As all the isolates of these species harbor T6SS (Figure 5), they probably encode effectors that belong to non-MIX classes.

Certain *Vibrionaceae* species appear to use MIX-effectors more than others. Many isolates of *Aliivibrio*, *V. coralliilyticus*, *V. azureus*, and *V. campellii* encode more than three MIX-effectors per genome, and were thus termed “professional MIXologists” (as they employ a cocktail of MIX-effectors). Strikingly, these strains encode the putative virulence MIX-effectors noted above. This suggests that these “professional MIXologists” adapted their MIX-secreting T6SSs to mediate not only antibacterial activities, but also their interactions with eukaryotes. Indeed, these species are known to interact with eukaryotes. *V. campbellii* [54], *V. coralliilyticus* [55], and *A. wodanis* [56] have been identified as pathogens of marine animals, whereas *A. fischeri* is not only a pathogen [57], it is also a symbiont of squid [5,58]. Recently, *A. fischeri* was reported to use T6SS to eliminate intra-species competitors within the squid light organ [24]. The authors focused their analysis on the effectors present in three T6SS auxiliary modules that contain VgrG (a T6SS-secreted component [12]). Our results expand their analysis, indicating that additional T6SS effectors (i.e., MIX-effectors) occur in the investigated genomes (e.g., *A. fischeri* MJ11). Thus, these additional MIX-effectors can explain the compatibility groups reported by Speare et al. Interestingly, *A. fischeri* also encodes a predicted virulence MIX-effector. It is tempting to speculate that this MIX-effector is used during pathogenicity against marine eukaryotes or during the symbiotic interaction with the squid.

Additional MIX-effectors, which were designated here as antibacterial, may function as trans-kingdom effectors that target both bacteria and eukaryotes, as has been demonstrated for the MIX-effector VasX [29,30]. Therefore, our analysis of MIX-effectors that target eukaryotes is probably a conservative under-estimation of the extent to which MIX-effectors are used by *Vibrionaceae* T6SSs to target eukaryotes. Moreover, additional non-MIX T6SS effectors [59] may also be used to target eukaryotes, and thus expand the breadth of *Vibrionaceae* T6SS utilization as a eukaryote-targeting determinant. Finally, we note that future experiments using animal models of infection or symbiosis are required to shed light on the roles of the predicted virulence MIX-effectors identified in this work.

### 2.5. Concluding Remarks

In this work, we employed a computational approach to characterize the MIX-effector repertoire in *Vibrionaceae* genomes. We identified 2342 MIX-effectors encoded by 1311 *Vibrionaceae* genomes (43.8% of the genomes we analyzed). The MIX-effectors were classified based on their polymorphic C-terminal toxin domains. There were 2315 MIX-effectors that were classified as antibacterial based on the presence of a putative downstream immunity gene. These antibacterial MIX-effectors carry predicted pore-forming, phospholipase, nuclease, peptidoglycan hydrolase, and protease activities. There were 27 MIX-effectors that were classified as virulence, anti-eukaryotic MIX-effectors, harboring protease, glycosyltransferase, pore-forming, and CNF1 predicted activities. Remarkably, the virulence MIX-effectors were found to be encoded by “professional MIXologists”, which are strains employing a cocktail of MIX-effectors. We hypothesize that these strains adapted their MIX-secreting T6SSs to mediate their interactions with eukaryotes, in addition to their role in interbacterial interactions. The collection of MIX-effectors presented here can serve as an excellent starting point for studying the contribution of specific effectors to the fitness of *Vibrionaceae* strains. Furthermore, the virulence MIX-effectors identified in this work can illuminate the role played by T6SSs in the interactions of *Vibrionaceae* with their eukaryotic hosts. Last but not least, the resources provided here can help in better understanding the catalytic mechanisms employed by secreted bacterial toxins.

## 3. Materials and Methods

### 3.1. Construction of Position-Specific Scoring Matrices (PSSMs) of Various MIX Domains

MIX sequences representing the various MIX clans [26] were retrieved from NCBI (Text S1). For each MIX sequence, five iterations of position-specific iterated BLAST (PSI-BLAST) against the reference protein database were performed (a maximum of 500 hits with a threshold of 10^−6^ were used in each iteration). The PSSMs were saved for use in reverse position specific BLAST (RPS-BLAST).

### 3.2. Identification of MIX-Effectors in Vibrionaceae

The protein sequences and feature tables of members of the *Vibrionaceae* family were downloaded from NCBI on 20 August 2018. A total of 2994 RefSeq genomes were analyzed. RPS-BLAST [33] was used to identify the MIX-containing proteins. The results were filtered using the following criteria: (i) expect values lower than 10^−6^, (ii) the alignment contained the core of the MIX motif (GxxY), (iii) the alignment length was at least 90 aa, (iv) the distance from the C-terminus was at least 100 aa (to remove proteins with no C-terminal extension), and (v) there was a lack of a signal peptide. In cases of overlap between various MIX domains (at least 80% overlap), the MIX domain with the lowest expected value was saved. Unique accessions located at the ends of the contigs were removed. Unique accessions appearing in the same organism in more than one contig were removed if the same downstream gene existed at the same distance.

The C-terminal domains of MIX-containing proteins were clustered using CD-HIT v4.17 [34]. The clustering threshold was set to 40% identity, and the sequences were clustered to the most similar cluster that met the threshold. The C-terminal domains were defined as the sequences located downstream of the MIX domains.

The proteins located downstream of the MIX-containing proteins on the same strand were defined as downstream proteins, and their distance from the MIX-containing proteins was calculated. The MIX-containing proteins and downstream proteins were analyzed using the NCBI Batch Conserved Domains-Search Tool [60]. Transmembrane topology and signal peptides were predicted using Phobius [61].

### 3.3. Construction of the Phylogenetic Tree

The DNA sequences of *rpoB* coding for DNA-directed RNA polymerase subunit beta were downloaded from NCBI on 25 August 2018. The partial sequences (less than 99% of the full-length gene) were removed. The sequences were aligned using MAFFT v7.408 FFT-NS-2 [62,63]. The evolutionary history was inferred using the neighbor-joining method [64]. The optimal tree with the sum of branch length equal to 8.19052967 is shown. The evolutionary distances were computed using the maximum composite likelihood method [65]. The analysis involved 2884 nucleotide sequences. The codon positions that were included were the first, second, third, and noncoding. All of the positions with less than 95% site coverage were eliminated. That is, fewer than 5% alignment gaps, missing data, and ambiguous bases were allowed at any position. There were a total of 4026 positions in the final dataset. Evolutionary analyses were conducted in MEGA7 [66]. The tree and additional datasets were visualized using iTOL [67].

BLASTP [33] was employed to identify the T6SS core components in the *Vibrionaceae* genomes. The proteins were aligned against representative proteins of *V. parahaemolyticus* RIMD 2210633 T6SS 1 and 2 (Table S1). The similarity was calculated by dividing the bit_score value by the query length multiplied by two. The minimal similarity threshold was 0.2. *Vibrionaceae* genomes, with at least three representative proteins that were marked as harboring T6SS (Dataset S4).

### 3.4. Strains and Media

*Escherichia coli* strain DH5α (λ pir) was used for routine cloning and plasmid amplification. The *E. coli* strain BL21 (DE3) was used for protein expression. *E. coli* were grown in 2xYT broth at 37 °C. The media were supplemented with Ampicillin (100 µg/mL) or kanamycin (30 µg/mL) when appropriate, so as to maintain the plasmids. The yeast strain that was used was *Saccharomyces cerevisiae* BY4741 (MATa, his3Δ0, leu2Δ0, met15Δ0, and ura3Δ0). Yeast were grown at 30 °C in a synthetic complete medium (synthetic drop-out media supplemented with uracil, leucine, tryptophan, histidine, and 2% glucose). To maintain plasmids, yeast were grown in selective synthetic complete media lacking leucine, and were supplemented with 2% glucose or 2% galactose and 1% raffinose as carbon sources.

### 3.5. Plasmids

The coding sequences of VPR01s_11_01580, VPR01s_11_01570, VPR01s_06_01710, and VPR01s_25_00650 were amplified from the *Vibrio proteolyticus* strain ATCC 15338 (also termed NBRC 13287) genomic DNA; the coding sequence of VIC_RS20535 was amplified from *Vibrio coralliilyticus* strain ATCC BAA-450 genomic DNA. For arabinose-inducible cytoplasmic expression in bacteria, the genes were inserted into the multiple cloning site of the pBAD/Myc–His vector (Invitrogen) harboring a kanamycin-resistance cassette [20] in-frame with the C-terminal *Myc*-6xHis tag. For periplasmic expression, the genes were inserted into the multiple cloning site of pPER5, a Kan^R^ pBAD/Myc–His in which the signal peptide of PelB was inserted into the NcoI/PstI sites, in-frame with the C-terminal *Myc*-6xHis tag. The Gibson Assembly method [68] was used for cloning into the bacterial expression vectors. The plasmids were transformed into *E. coli* using electroporation.

For the galactose-inducible expression in yeast, the genes were inserted into the multiple cloning site of the shuttle vector pGML10 (Riken) using restriction cloning, in-frame with the C-terminal *Myc* tag. The yeast transformations were performed using the LiAc method, as previously described [69].

### 3.6. Bacterial Growth Assays

*E. coli* BL21 (DE3) containing arabinose-inducible expression vectors were grown overnight in 2xYT broth supplemented with kanamycin (30 µg/mL) and glucose (0.2%). The cultures were washed twice with 2xYT broth, and normalized to an OD_600_ = 0.01 in 2xYT broth supplemented with kanamycin (30 µg/mL). The cultures were then transferred to 96-well plates (200 µL per well) in triplicates. The cultures were grown at 37 °C in a BioTek SYNERGY H1 microplate reader with continuous shaking at 205 cpm. OD_600_ readings were acquired every 10 min. After 2 h, L-arabinose was added to each well to a final concentration of 0.05% so as to induce protein expression. The experiments were performed three times with similar results.

### 3.7. Yeast Toxicity

BY4741 yeast strains containing pGML10 expression plasmids were streaked onto synthetic dropout plates lacking leucine and containing either 2% glucose (which represses expression from plasmids) or 2% galactose and 1% raffinose (for protein induction). The plates were incubated at 30 °C for two days.

### 3.8. Protein Expression

For expression in *E. coli*, BL21 (DE3) cells containing arabinose-inducible expression vectors were grown overnight in 2xYT broth supplemented with kanamycin (30 µg/mL) and glucose (0.2%). The cultures were washed twice with 2xYT broth and then normalized to an OD_600_ of 0.5 in 2xYT broth supplemented with kanamycin (30 µg/mL) and L-arabinose (0.05%). The cultures were incubated at 37 °C for 3 h. For each culture, 0.5 OD_600_ units were pelleted and resuspended in a 50 µL Tris-glycine SDS sample buffer x2 (Novex, Life Sciences).

For the galactose-inducible expression in yeast, the cells were grown overnight at 30 °C in glucose-containing (2%) media. The cells were washed twice and resuspended in galactose and raffinose-containing media (2% and 1%, respectively) at OD_600_ = 1.0. Next, the cells were grown at 30 °C for 6 h. Yeast cell lysis on 1.0 OD_600_ units was performed as previously reported [49,69].

The proteins from the total cell lysates were resolved on SDS–polyacrylamide gel electrophoresis and transferred to nitrocellulose membranes. Protein expression was detected using anti-myc antibodies (1:1000 dilution) (9E10, SC-40, Santa Cruz). Loading of the total protein lysate was visualized using Ponceau S-staining of the membranes.

### 3.9. Multiple Sequence Alignments and Secondary Structure Predictions

The sequence alignments were extracted from the HHpred results [36] or were performed using Clustal Omega [70]. Secondary structure predictions were performed using Jpred4 [71]. The alignments were visualized in Jalview [72].

## Figures and Tables

**Table 1:** Summary of the predicted activities of the *Vibrionaceae* MIX--effectors’ C-terminal toxin domains.

Predicted Toxic Activity	Occurrences	MIX Clans	Predicted Antibacterial (AB)/Virulence (Vir)
Pore-forming	835	I, II, IV, IV + II, V + I, V + IV	AB
VP1388 homologs	800	I	AB
Nuclease	331	V, V + I	AB
Truncated (no C-terminal toxin domain)	124	I, IV, V, V + IV	-
DUF2335 (phospholipase)	121	V, V + I	AB
Peptidoglycan hydrolase	35	V	AB
Peptidase	6	V	AB
Nucleotide deaminase	2	I	AB
Unknown	61	I, IV, V, V + IV, V + I + IV	AB
Protease	15	V	Vir
Glycosyltransferase	5	V	Vir
Pore-forming	2	I	Vir
CNF1	1	V	Vir
Unknown	4	V	Vir

**Table 2:** Predicted virulence MIX-effector clusters.

Cluster	Accessions	Bacterial Species	Predicted Activity/Toxin Domain
83	WP_040902815.1	*V. proteolyticus*	CNF1 deamidase
27	WP_065611703.1, WP_061012685.1, WP_060992952.1, WP_017020872.1, WP_061036948.1, WP_061012685.1, WP_105064022.1, WP_061029312.1, WP_061004481.1, WP_023604334.1	*A. wodanis,* *A. logei,* *A. sifiae*	Protease
55	WP_005429108.1, WP_052438057.1	*V. campbellii*	Cysteine protease
53	WP_012535377.1, WP_063646315.1	*A. fischeri*	Peptidase
58	WP_021709833.1, WP_052035761.1	*V. proteolyticus*, *V. azeurus*	Unknown
38	WP_006962196.1, WP_043008000.1, WP_095560114.1, WP_099609290.1, WP_064487344.1	*V. coralliilyticus*	Glycosyltransferase
72	WP_073603189.1	*V. aerogenes*	Pore-forming
76	WP_073605246.1	*V. aerogenes*	Pore-forming

## Supplementary Materials

The following are available online at http://www.mdpi.com/1660-3397/16/11/433/s1, Figure S1: multiple sequence alignments of predicted virulence MIX-effectors. Figure S2: immunoblot of MIX-effectors’ expression in bacteria and yeast. Text S1: MIX sequences representing various MIX clans. Table S1: representative proteins of *V. parahaemolyticus* RIMD 2210633 T6SSs 1 and 2. Dataset S1: *Vibrionaceae* genomes included in the analysis. Dataset S2: MIX-effectors and downstream proteins identified in the analysis. Dataset S3: summary of clusters of MIX-effectors and their predicted activities. Dataset S4: summary of T6SS core components and MIX-effectors in *Vibrionaceae* genomes.
