# Supplementary material for: The Antibacterial and Anti-Eukaryotic Type VI Secretion System MIX-Effector Repertoire in Vibrionaceae
Source: Mar Drugs. 2018 Nov 4;16(11):433. doi: 10.3390/md16110433 (PMC6267618; doi:10.3390/md16110433)
Supplement: Supplementary file 1 [file marinedrugs-16-00433-s001.zip › Figure_S1.pdf]

a

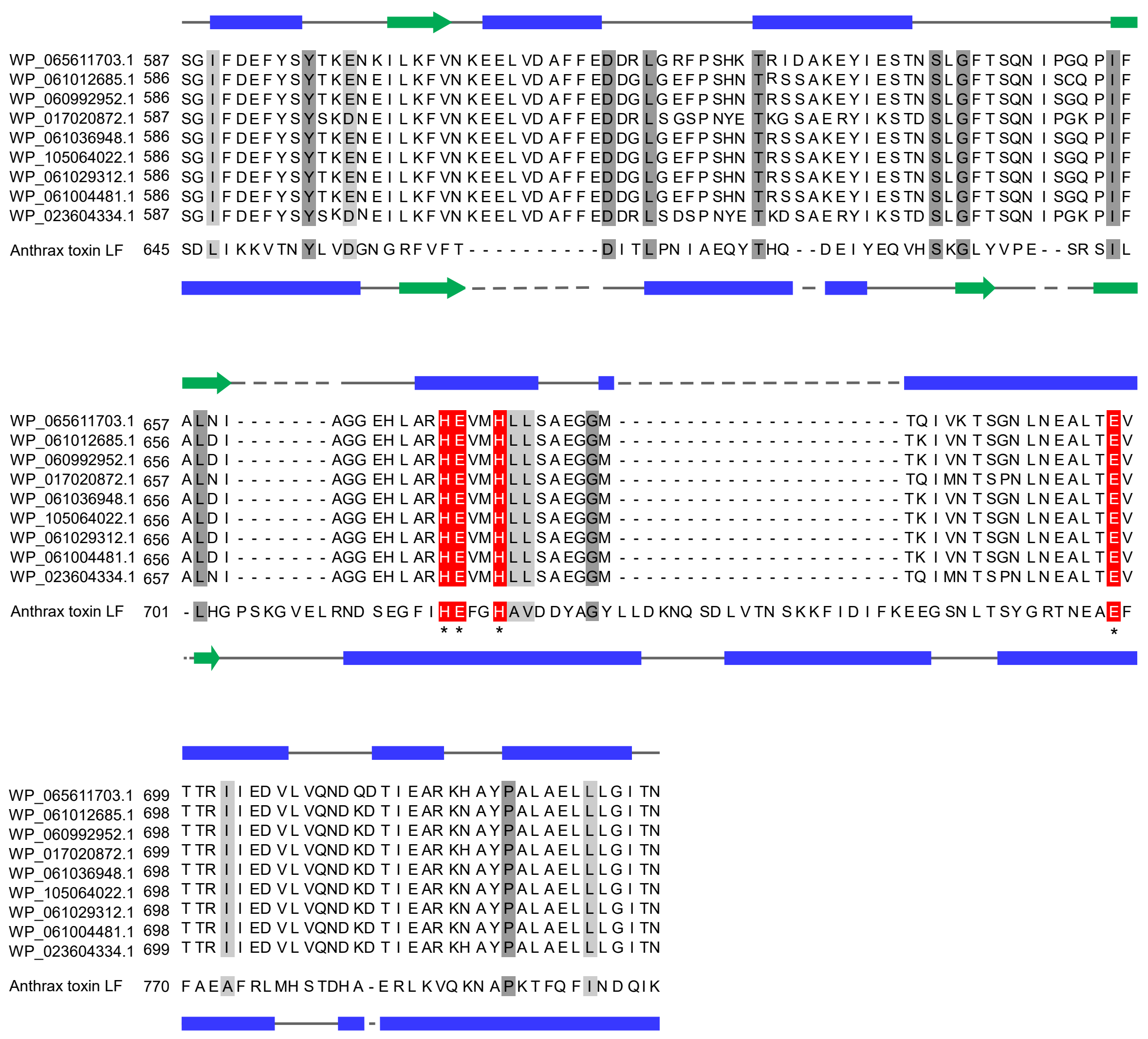
WP_065611703.1 587 SGIFDEFYSYTKENKILKFVNKEELVDAFFEDDRLGRFPSHKTRIDAKEYIESTNSLGFTSQNIPGQPIF
WP_061012685.1 586 SGIFDEFYSYTKENEILKFVNKEELVDAFFEDDGLGEFPSHNTRSSAKEYIESTNSLGFTSQNISCQPIF
WP_060992952.1 586 SGIFDEFYSYTKENEILKFVNKEELVDAFFEDDGLGEFPSHNTRSSAKEYIESTNSLGFTSQNISGQPIF
WP_017020872.1 587 SGIFDEFYSYSKDNEILKFVNKEELVDAFFEDDRLSGSPNYETKGSAERYIKSTDSLGFTSQNIPGKPIF
WP_061036948.1 586 SGIFDEFYSYTKENEILKFVNKEELVDAFFEDDGLGEFPSHNTRSSAKEYIESTNSLGFTSQNISGQPIF
WP_105064022.1 586 SGIFDEFYSYTKENEILKFVNKEELVDAFFEDDGLGEFPSHNTRSSAKEYIESTNSLGFTSQNISGQPIF
WP_061029312.1 586 SGIFDEFYSYTKENEILKFVNKEELVDAFFEDDGLGEFPSHNTRSSAKEYIESTNSLGFTSQNISGQPIF
WP_061004481.1 586 SGIFDEFYSYTKENEILKFVNKEELVDAFFEDDGLGEFPSHNTRSSAKEYIESTNSLGFTSQNISGQPIF
WP_023604334.1 587 SGIFDEFYSYSKDNEILKFVNKEELVDAFFEDDRLSDSPNYETKDSAERYIKSTDSLGFTSQNIPGKPIF
Anthrax toxin LF 645 SDLIKKVTNYLVDGNGRFVFT----------DITLPNIAEQYTHQ--DEIYEQVHSKGLYVPE--SRSIL
WP_065611703.1 657 ALNI-------AGGEHLARHEVMHLLSAEGGM--------------------TQIVKTSGNLNEALTEV
WP_061012685.1 656 ALDI-------AGGEHLARHEVMHLLSAEGGM--------------------TKIVNTSGNLNEALTEV
WP_060992952.1 656 ALDI-------AGGEHLARHEVMHLLSAEGGM--------------------TKIVNTSGNLNEALTEV
WP_017020872.1 657 ALNI-------AGGEHLARHEVMHLLSAEGGM--------------------TQIMNTSPNLNEALTEV
WP_061036948.1 656 ALDI-------AGGEHLARHEVMHLLSAEGGM--------------------TKIVNTSGNLNEALTEV
WP_105064022.1 656 ALDI-------AGGEHLARHEVMHLLSAEGGM--------------------TKIVNTSGNLNEALTEV
WP_061029312.1 656 ALDI-------AGGEHLARHEVMHLLSAEGGM--------------------TKIVNTSGNLNEALTEV
WP_061004481.1 656 ALDI-------AGGEHLARHEVMHLLSAEGGM--------------------TKIVNTSGNLNEALTEV
WP_023604334.1 657 ALNI-------AGGEHLARHEVMHLLSAEGGM--------------------TQIMNTSPNLNEALTEV
Anthrax toxin LF 701 -LHGPSKGVELRNDSEGFIHEFGHAVDDYAGYLLDKNQSDLVTNSKKFIDIFKEEGSNLTSYGRTNEAEF
* * * *
WP_065611703.1 699 TTRIIEDVLVQNDQDTIEARKHAYPALAELLLGITN
WP_061012685.1 698 TTRIIEDVLVQNDKDTIEARKNAYPALAELLLGITN
WP_060992952.1 698 TTRIIEDVLVQNDKDTIEARKNAYPALAELLLGITN
WP_017020872.1 699 TTRIIEDVLVQNDKDTIEARKHAYPALAELLLGITN
WP_061036948.1 698 TTRIIEDVLVQNDKDTIEARKNAYPALAELLLGITN
WP_105064022.1 698 TTRIIEDVLVQNDKDTIEARKNAYPALAELLLGITN
WP_061029312.1 698 TTRIIEDVLVQNDKDTIEARKNAYPALAELLLGITN
WP_061004481.1 698 TTRIIEDVLVQNDKDTIEARKNAYPALAELLLGITN
WP_023604334.1 699 TTRIIEDVLVQNDKDTIEARKHAYPALAELLLGITN
Anthrax toxin LF 770 FAEAFRLMHSTDHA-ERLKVQKNAPKTFQFINDQIK

b

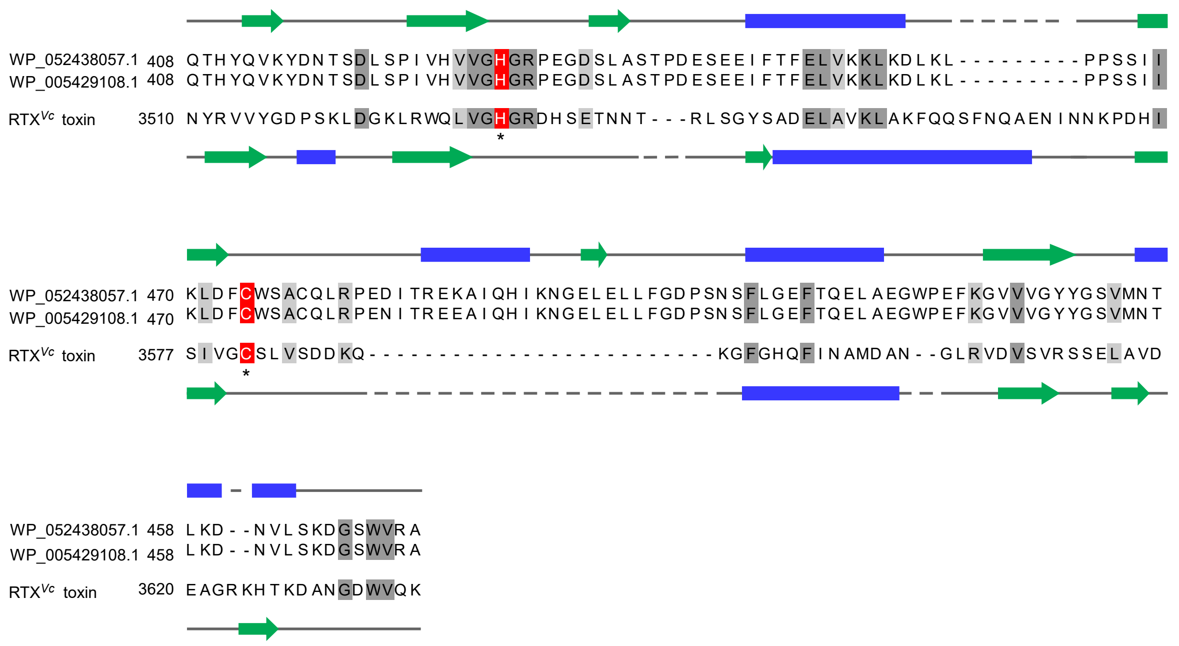
WP_052438057.1 408 QTHYQVKYDNTSDLSPIVHVVGHGRPEGDSLASTPDESEEIFTFELVKKLKDLKL---------PPSSII
WP_005429108.1 408 QTHYQVKYDNTSDLSPIVHVVGHGRPEGDSLASTPDESEEIFTFELVKKLKDLKL---------PPSSII
RTX^{Vc} toxin 3510 NYRVVYGDPSKLDGKLRWQLVGHGRDHSETNNT---RLSGYSADELAVKLAKFQQSFNQAENINNKPDHI
*
WP_052438057.1 470 KLDFCWSACQLRPEDITREKAIQHIKNGELELLFGDPSNSFLGEFTQELAEGWPEFKGVVVGYYGSVMNT
WP_005429108.1 470 KLDFCWSACQLRPENITREEAIQHIKNGELELLFGDPSNSFLGEFTQELAEGWPEFKGVVVGYYGSVMNT
RTX^{Vc} toxin 3577 SIVGCSLVSDDKQ-----------------------KGFGHQFINAMDAN--GLRVDVSVRSSELAVD
*
WP_052438057.1 458 LKD--NVLSKDGSWVRA
WP_005429108.1 458 LKD--NVLSKDGSWVRA
RTX^{Vc} toxin 3620 EAGRKHTKDANGDWVQK

c

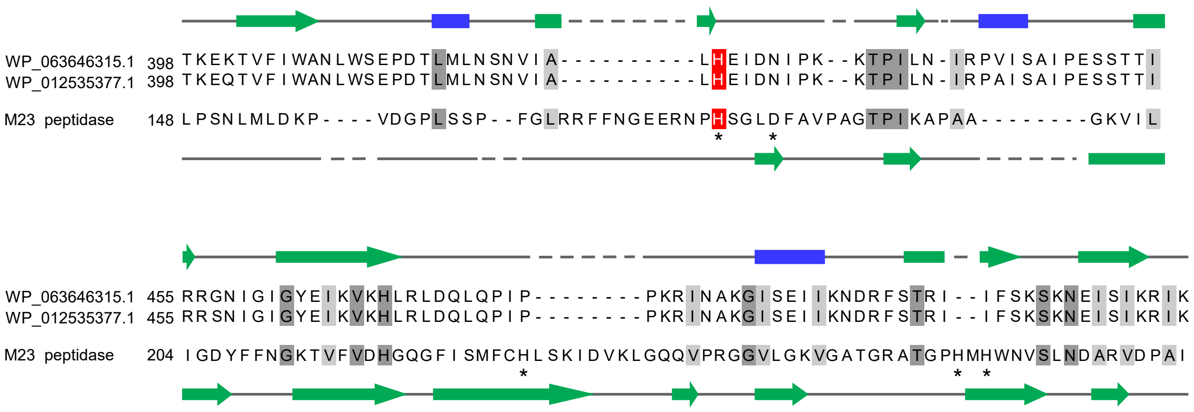

WP_063646315.1 398 TKEKTVFIWANLWSEPDTLMLNSNVIA----------LHEIDNIPK--KTPILN-IRPVISAIPESSTTI
WP_012535377.1 398 TKEQTVFIWANLWSEPDTLMLNSNVIA----------LHEIDNIPK--KTPILN-IRPAISAIPESSTTI
M23 peptidase 148 LPSNLMLDKP----VDGPLSSP--FGLRRFFNGEERNPHSGLDFAVPAGTPIKAPAA--------GKVIL
WP_063646315.1 455 RRGNIGIGYEIKVKHLRLDQLQPIP--------PKRINAKGISEIIKNDRFSTRI--IFSKSKNEISIKRIK
WP_012535377.1 455 RRSNIGIGYEIKVKHLRLDQLQPIP--------PKRINAKGISEIIKNDRFSTRI--IFSKSKNEISIKRIK
M23 peptidase 204 IGDYFFNGKTVFVDHGQGFISMFCHLSKIDVKLGQQVPRGGVLGKVGATGRATGPHMHWNVSLNDARVDPAI

d

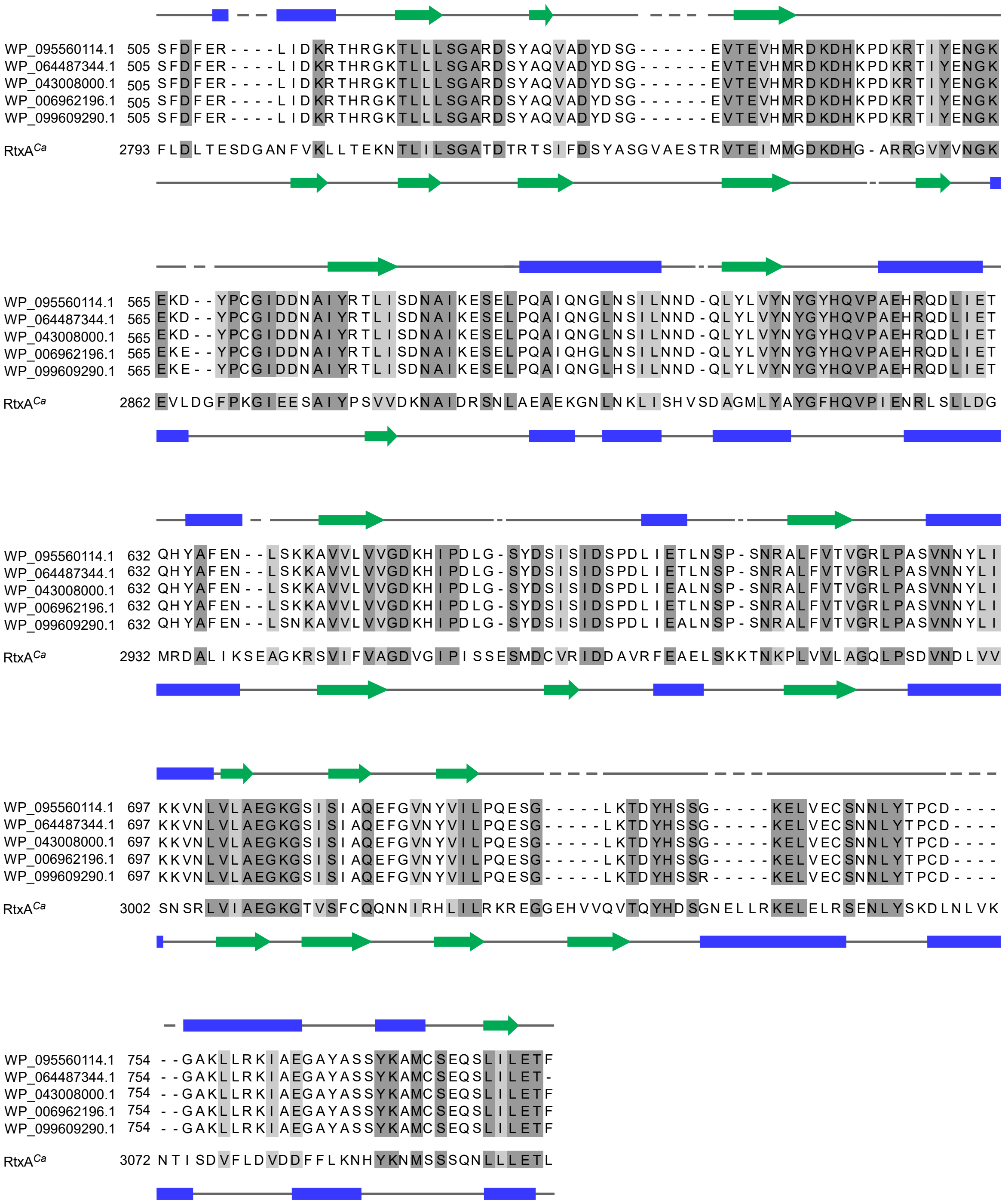

WP_095560114.1 505 SFDFER----LIDKRTHRGKTLLLSGARDSYAQVADYDSG------EVTEVHMRDKDHKPDKRTIYENGK
WP_064487344.1 505 SFDFER----LIDKRTHRGKTLLLSGARDSYAQVADYDSG------EVTEVHMRDKDHKPDKRTIYENGK
WP_043008000.1 505 SFDFER----LIDKRTHRGKTLLLSGARDSYAQVADYDSG------EVTEVHMRDKDHKPDKRTIYENGK
WP_006962196.1 505 SFDFER----LIDKRTHRGKTLLLSGARDSYAQVADYDSG------EVTEVHMRDKDHKPDKRTIYENGK
WP_099609290.1 505 SFDFER----LIDKRTHRGKTLLLSGARDSYAQVADYDSG------EVTEVHMRDKDHKPDKRTIYENGK
RtxA^{Ca} 2793 FLDLTESDGANFVKLLTEKNTLILSGATDTRTSIFDSYASGVAESTRVTEIMMGDKDHG-ARRGVYVNGK
WP_095560114.1 565 EKD--YPCGIDDNAIYRTLISDNAIKESELPQAIQNGLNSILNND-QLYLVYNYGYHQVPAEHRQDLIET
WP_064487344.1 565 EKD--YPCGIDDNAIYRTLISDNAIKESELPQAIQNGLNSILNND-QLYLVYNYGYHQVPAEHRQDLIET
WP_043008000.1 565 EKD--YPCGIDDNAIYRTLISDNAIKESELPQAIQNGLNSILNND-QLYLVYNYGYHQVPAEHRQDLIET
WP_006962196.1 565 EKE--YPCGIDDNAIYRTLISDNAIKESELPQAIQHGLNSILNND-QLYLVYNYGYHQVPAEHRQDLIET
WP_099609290.1 565 EKE--YPCGIDDNAIYRTLISDNAIKESELPQAIQNGLHSILNND-QLYLVYNYGYHQVPAEHRQDLIET
RtxA^{Ca} 2862 EVLDGFPKGIEESAIYPSVVDKNAIDRSNLAEAEKGNLNKLISHVSDAGMLYAYGFHQVPIENRLSLLDG
WP_095560114.1 632 QHYAFEN--LSKKAVVLVVGDKHIPDLG-SYDSISIDSPDLIETLNSP-SNRALFVTVGRLPASVNNYLI
WP_064487344.1 632 QHYAFEN--LSKKAVVLVVGDKHIPDLG-SYDSISIDSPDLIETLNSP-SNRALFVTVGRLPASVNNYLI
WP_043008000.1 632 QHYAFEN--LSKKAVVLVVGDKHIPDLG-SYDSISIDSPDLIEALNSP-SNRALFVTVGRLPASVNNYLI
WP_006962196.1 632 QHYAFEN--LSKKAVVLVVGDKHIPDLG-SYDSISIDSPDLIETLNSP-SNRALFVTVGRLPASVNNYLI
WP_099609290.1 632 QHYAFEN--LSNKAVVLVVGDKHIPDLG-SYDSISIDSPDLIEALNSP-SNRALFVTVGRLPASVNNYLI
RtxA^{Ca} 2932 MRDALIKSEAGKRSVIFVAGDVGIPISSESMDCVRIDDAVRFEAELSKKTNKPLVVLAGQLPSDVNDLVV
WP_095560114.1 697 KKVNLVLAEGKGSISIAQEFGVNYVILPQESG-----LKTDYHSSG-----KELVECSNNLYTPCD----
WP_064487344.1 697 KKVNLVLAEGKGSISIAQEFGVNYVILPQESG-----LKTDYHSSG-----KELVECSNNLYTPCD----
WP_043008000.1 697 KKVNLVLAEGKGSISIAQEFGVNYVILPQESG-----LKTDYHSSG-----KELVECSNNLYTPCD----
WP_006962196.1 697 KKVNLVLAEGKGSISIAQEFGVNYVILPQESG-----LKTDYHSSG-----KELVECSNNLYTPCD----
WP_099609290.1 697 KKVNLVLAEGKGSISIAQEFGVNYVILPQESG-----LKTDYHSSR-----KELVECSNNLYTPCD----
RtxA^{Ca} 3002 SNSRLVIAEGKGTVSFCQQNNIRHLILRKREGGEHVVQVTQYHDSGNELLRKELELRSENLYSKDLNLVK
WP_095560114.1 754 --GAKLLRKIAEGAYASSYKAMCSEQSLILETF
WP_064487344.1 754 --GAKLLRKIAEGAYASSYKAMCSEQSLILET-
WP_043008000.1 754 --GAKLLRKIAEGAYASSYKAMCSEQSLILETF
WP_006962196.1 754 --GAKLLRKIAEGAYASSYKAMCSEQSLILETF
WP_099609290.1 754 --GAKLLRKIAEGAYASSYKAMCSEQSLILETF
RtxA^{Ca} 3072 NTISDVFLDVDDFFLKNHYKNMSSSQNLLLETL

e

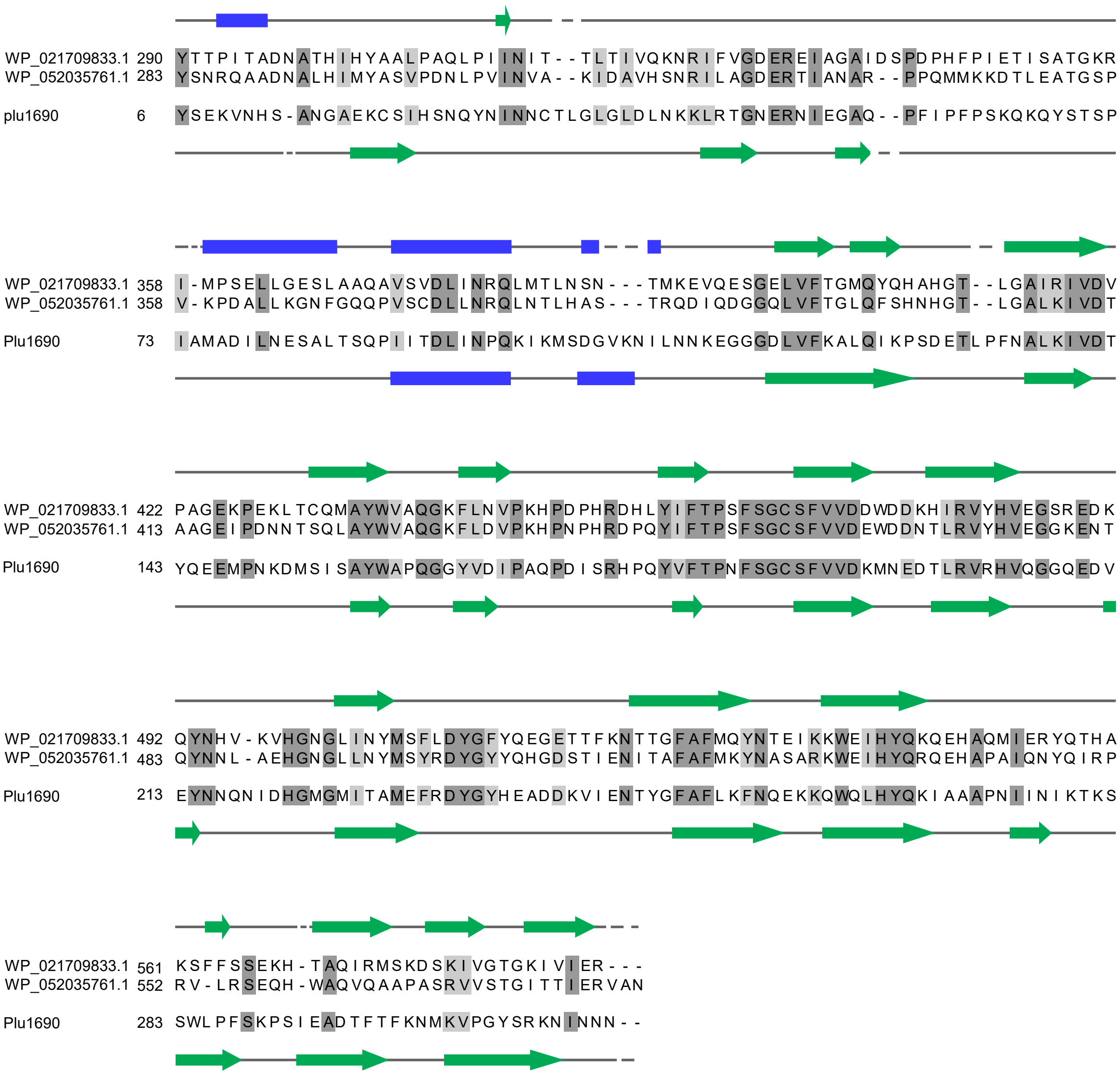

Figure S1. C-terminal domains of predicted virulence MIX-effectors are homologous to other virulence toxins. (**a-e**) Multiple sequence alignments of predicted virulence MIX-effectors and their homologous toxins with the start positions indicated. MIX-effector C-termini of clusters 27 (a), 55 (b), 53 (c), 38 (d), and 58 (e) are shown above the sequence of the virulence homologs: Bacillus anthracis anthrax toxin lethal factor (WP_001022097.1; PDB: 5d1u) (a), *Vibrio cholerae* RtxA toxin cysteine protease domain (NP_231094.1; PDB: 3gcd) (b), *Pseudomonas* M23 family peptidase (WP_003092745.1; PDB: 2hsi) (c), *Chromobacterium amazonense* RtxA (WP_083340985.1) (d), and *Phtorhabdus* Plu1690 (CAE13983.1) (e). Catalytic residues of virulence homologs are denoted by an asterisk. Catalytic residues that are conserved in MIX-effectors are in red. Other identical residues are in dark gray, whereas residues that share similar chemical properties are in light gray. The predicted secondary structure (Jpred4) of the MIX-effector sequences are depicted above the alignments, whereas secondary structures of homologous sequences were taken from PDB-deposited structures for (a-c) or predicted (Jpred4). Green arrows represent beta sheets and blue rectangles represent alpha helices. Gaps in sequences are denoted in the secondary structures by dashed lines. Alignments were performed using HHpred (a-c) or Clustal Omega (d-e).
