# Supplementary material for: The Antibacterial and Anti-Eukaryotic Type VI Secretion System MIX-Effector Repertoire in Vibrionaceae
Source: Mar Drugs. 2018 Nov 4;16(11):433. doi: 10.3390/md16110433 (PMC6267618; doi:10.3390/md16110433)
Supplement: Supplementary file 1 [file marinedrugs-16-00433-s001.zip › Table_S1.docx]

**Table S1 – Representative proteins of *V. parahaemolyticus* RIMD 2210633 T6SS 1 and 2**

| **System** | **Protein Accession** | **Description** |
| --- | --- | --- |
| T6SS 1 | NP_797781.1 | VP1402 (TssB) |
| T6SS 1 | NP_797782.1 | VP1403 (TssC) |
| T6SS 1 | NP_797785.1 | VP1406 (TssG) |
| T6SS 1 | NP_797783.1 | VP1404 (TssE) |
| T6SS 1 | NP_797784.1 | VP1405 (TssF) |
| T6SS 1 | NP_797791.1 | VP1412 (TssJ) |
| T6SS 1 | NP_797792.1 | VP1413 (TssK) |
| T6SS 1 | NP_797772.1 | VP1393 (Hcp/TssD) |
| T6SS 1 | NP_797773.1 | VP1394 (VgrG/TssI) |
| T6SS 2 | NP_800545.1 | VPA1035 (TssB2) |
| T6SS 2 | NP_800544.1 | VPA1034 (TssC2a) |
| T6SS 2 | NP_800543.1 | VPA1033 (TssC2b) |
| T6SS 2 | NP_800539.1 | VPA1029 (TssG2) |
| T6SS 2 | NP_800541.1 | VPA1031 (TssE2) |
| T6SS 2 | NP_800540.1 | VPA1030 (TssF2) |
| T6SS 2 | NP_800552.1 | VPA1042 (TssJ2) |
| T6SS 2 | NP_800551.1 | VPA1041 (TssK2) |
| T6SS 2 | NP_800537.1 | VPA1027 (Hcp2) |
| T6SS 2 | NP_800536.1 | VPA1026 (VgrG2) |
