# Supplementary material for: The Antibacterial and Anti-Eukaryotic Type VI Secretion System MIX-Effector Repertoire in Vibrionaceae
Source: Mar Drugs. 2018 Nov 4;16(11):433. doi: 10.3390/md16110433 (PMC6267618; doi:10.3390/md16110433)
Supplement: Supplementary file 1 [file marinedrugs-16-00433-s001.zip › Text_S1.docx]

**Text S1 - MIX sequences representing MIX I-V clans**

MIX I - *Vibrio parahaemolyticus* VP1388 (WP_011105821.1)

MWRIQMHLVCKFIPSSKLSSNELSYVLTPDECIGQLSRVRNSEDILRNLPKELAQKISISAKKSSSGLLTAIRHELGNGNWVALSSFSRRTPLTDTQLQSFPRLKAQLESVSSTGESKVYKAGYKQVKDDVTLVRSYTHVPSEPSPDQKIVVEFAGQWSSNAACLMLGKTEAQKEKVTVGKADTENKHRSLATFKDLEAEGKTLYIKIPCSDQPQPILLKLAEDLQPVDKETQMDEWDNVLVPVVPLHFPGSDKSDEAAEVFKSGYVYVVWNNKIWREVAITENGYFSDTDINSVREGSRPKRHADIYMTNPETGGVFAYEPFQIVQNGKVVSEGSLNGSGEARVFNLVEEEVEIVMTGYEPQIKEKIETNLSPINASSPVGRSAQGYPLPHIWLPYKIKGEPQEVYLAYNSKRLSESELSELESDPGTKAIKVTDLNHYSSEKSFKMGDGSVRLLSVLPSAATSKPEKYAMLRSQINKNVAVVYLLKSVEIVFEYPGYTTLDESDDYFELRQSDGDWSQRVCLRQCIKKENGSRLIRFTGWPAEVKEVDLLRGYQGNSHHGRDNKTVIFAQTPIADLLAYKKKDQPS

MIX II - *Proteus mirabilis* IdsD (WP_020946444.1)

MTGEVNEKYLTPQERKARQMVKAVNEASPRNLPADAVVCPCENEHRPVYPVRYAYTNFYCDLHFSTIEQAPNKTLEASIPPSINQLLNAKDVTASKGFSARLLRQGWVYVFEEGNYPTRSNSSNKSYQEQNVDATKGRLLVFQHQVTTSDGNENFIPYIFKQLKNGGVTLKKNGNSNPYLAIPKDVKEATILFSESKLSDYTLKKIISSSKFRSKLMQKINFIDYNNNDYCIELNKDNLNRLVEDYKEEVDKFKLFVKEFTHSNIPSSFFSDTTKIPDLPQDATVLINQVNSVLDYNEKATLLILKDPVGYQKDILSYYNIVTKLHLLYQNYYSHPDKIGQFITSIQEASHHIKDTDEKEKMQTILKESINQNALDNEWKNIHKTFIFFEKHQRIVLSLYESFMNNPAIINENGGLKHYFDYAFSYHERITKEDVFSIDFFKDLNQAFDLYFDLVSPLMNSIEGQRTLDKLYSINDEENNSLWVGVTKKVISLIANSKIKDALLNVQEYAMHIENFVNKLAFICSDSIGFAFTKTSKVLSHYDIKNRLINTKGIDYLAQKILPMILAFCNTKISLTEFVKLSGNELNQWMEQLRKLTGQIVPNLQHPKLNKLFSWKQKIINLGEETAVLIPKIEIIDITKNKIYIYGKDALQVSTKLFLNGFSMITGSIQAYTLQGMSLYERNDPLKLSPYNLYTAQIIANLFVASYSILKVSQEATKLSQTVSSTTLKFFLDKIKLPMLTTEVGTKRMAALGKIAGAVGAALAARDALEAFHIGNYKQSVSNIAIVIGSIILITAVTGGWALFAGALILGGFISSQLTSWSHLETLLKHSFWGNEKRSNFWDNDRPTPIGEQLKQYIKEFEFYKQKGLIELQEFYNLFYTAKMTQEKIPNGKLRLSFEFTNFTPGISEVYFHFVTEVGYHSGLAEEIKTPSSAYVLNKRKDLLEISEQLKMASEKGDWNPETGIFKFSLEVQSQLVNTYSAFGAHPNSRIGIEDLYWYYQVNPEVTTPMRYINWGGDTQENNQLLGFINSENI

MIX III - *Burkholderia thailandensis* BTH_I2691 (WP_009891761.1)

MAGSDDRCDVCKLQGLAVYPSRYAVVPKTFDAPALGPFDDKSVTGVALTQSKYGLRQLREGFVYLLYESGPRGPFHWEVYSVAPDGTLWKQLDTASVKRVSAPQACQRKGHSASRVQYLVIEKPHQCGNVWIAFSEWPWSQDTVKRYGGNDANAASLRKKRMQLIEPSKWISAPKPGPYSAPLTEANLQRVIEHAPAVATTAKGEPVGLLSTELPEAVSYGDLGAFRDARLQICTSRYPWTMRNKAAAGAKLSPAAETVKHAEGSTRNAKGAPCVPMMVALWDGIGITHELNGFRNDAQARFLQYCDEQALRINALQWIDQAKLAVEAGARRKATFDHSPATPGSTSWYYPDAVAKQKAAAKTPADQRFWSDYQWMGENGVPPSYARQITQFRAAPSSPAYQDAMARARKYVEAKPRIEQDRAKKIDQDTVTGTEHDWAKYREKLADDTSGKKGASNGNVKRIDVFRILYQRIQDQKQALLAQRTADVANWLDAPLFLAALEDYHEENGFDGIAFEFAIFHAIVGLSAERSGVAALNKLIDRLDPTKPESLVWRMVASNQKKSKEALKLALAKAEANKNVVLETVGEGFNVFAETSEKLKKFAEFYTKMDEMSKQAKQLNAIDQAMHDKGVDKVVVSVGHLVFQRFPYKGADLMGLGNGVSEAIIKSVFMLRVGIKSEEVKDLVLQQAKVEPQLRSKFLGEYQAQRRMGRSSPEAFRMATSAVAKTEGSNVLANRWSKLKVRESVGLVGVIGLIEVIGLIKLINKSDKEGRDWAELGGACFSVAGAAAEVALKPLEAFKFEEGAKNLKIFGGYCGALAAGVGTTLDTLDAIEKWGSGERLVSVLYFMKGMLGFGTTMAFGLTALSSSAPVFERLASNITGRQIRIVILDRIGQGITDAAARKVAIAAGERVATVAAERVGAMVVGRGLLIMASWQIQVAIISLQFVIWYFGNDDLQNSIKDSIFGVGGKYKNTEDLKKQNEAFDKALVAVGFKDDTASDKEEAKSHR

MIX IV - *Vibrio cholerae* VCA0020 (WP_000070352.1)

MSNPNQAAKTGQTNDAQNPASACPFKQPLIGIIPVRYAFDVYDDQGQALHPLPKADRQWKGQFSIKQRSYTLRQLRDGWLYVYDETAKTLHEYEVVGCKLTKIDWSDDEANKPTHERGSKGESKSCLLYPAQHTLSIGYAHQRWTWRVCEHMRSNTSSRHAVMRKVSLKQFESNGTHPHAHFAQYLEDYVADIGTPAEQDIFKDTCTPSLPVEKSEEAVKGTEFKFVADKAVVSSSDYLQDLPEQNCGLFVALNDPLADVSDLFVTFTTQVAKRTKAIGDETQQHKMQMAELTRTLGRIRLEEKEIPDFVKQDPIRILELERAITEYCATAKLAEIESHHLASEGHSPSGNYALMQQQAEQKLAELKTLYRFEPTSAQMRKWRKKDNSFIDEVRWADLDNFLVEHYTELKGLDEQIKQHYAQFMSAFNQLGLDPLLFGMDNQDEVQQAYLLALTSQFLVVVTQVNHDEKSLEILKKDLSFDSPKNLMALASTGFSLQANQAINNHIQGFSTAFLSTSNPSDMVAFATAIANWDTFTGDERIQEKAWFKRWIEPAQSSFGALQKAVANQAKESWQAVMELLFPYQNQPKGGTPSLLANLRLLLVESLVREEAVLQHNPKYAAELKQFETKLNAILQEMNDALELKPGNVSPKNHQIATAQSAQRKLGQLLSSELPMMLTLKNQAAMNTFQQSVNEKLSALSKNVKTSSASVSQKLGGLGGLLFALNLWNTMTVLENIRYKVAQYPSWNPFKNPALGEAIYATGNTIVVAGAISAGRAWVTIAEQGLLDRTLKNALNTTKVLGTKDALKTFAKSIALVATVGMIASALETWESWGKFNDSSKTDLERFGYLLKAGATGAQGIIFYIQFFTLLGSGIGGPSIAAISAGWMLAGFAVIGIVYLIGVILTNVFKRSELEIWLSKSTWGKESAHWPVGKELTELEHLLHRPSLRLSQVTQRKAAQWMDSGSLQWQLELTLPDYLKGQTIGLQITRLPAQPAYYQPQREAVTPILINEQQGKWSIEDNQPVYRITLGGSEKDTVGVCVALPLRWGKELSLKFYASGTRAGELDLQSAEANDIATRNLVVGKG

MIX V - *Vibrio parahaemolyticus* VPA1263 (WP_011106455.1)

MSYKVLPLSEVMSHEFGLIEGNFHNISEADIASAIPSHLTFEQLKQHLYEGNLALVSDTPQTPALLSYNDPIGPKTWRLNSEVISELSDDAANNLLAITKTTRATGGYRSCIGETSTLERVYTPQPIATESEEEITKEFEYSFEVGCSDATIKKMVHSDFALAKTEKENAVTRWEQTNTEQGTRYTALCVFDEPKRLNIHIADDNLGLTPLEAVTLQKAGSCKTDEGFIPVVPAVRLGERLGLPTEGYYYHFNDGELVQEYKILGEEKWAFYATQSTQDKLNDERGFTKDQSAILVYWKLADQIIENQYIIYLERQITREELDNLSEDWLCENGIKLDIPALFDAVKQPEEARSEGNNDETQAEQTAATHTVTVGEDWQSIAELYGMGAKALLLLNPVFEADPLSLAVGDEIIVAEKQQQQAPDKKNTFPPLRPQTYNNIRNSHYQHSDPLLGLTKYRAINTADCIEGDVVILNLKDATSSIVFAKSCTRPEGCIEIGDQQESISNFGPWSFFFAQANANPAAVIPAIQATQAQMAMGSSAAVAGSPEQMQQTQTAAMQLDKLAGTLKEKIVEGYRWQVEGIGALFAMQQSLFGDNTQYTDQDLRQVTTAQSRVRVHITEPQGGEFYPHVQGYHVDDTRIPIKYVKQGNNGQLSVAIEENGPTIYWTPEENGEASWQTTPDHSDGFEKDDILVTPIHSDSDANVTVTPAPEEKDWRDAILVFPESSGIAPLYVVYKESPRDKPGVVTGKGEDIFGIWLADAGKDLGAPIPSQIADKLRGREFSSFDAFREGFWYAVGEDQTLINQFSRANQRLIKRGRSPFSLPSEQVGGRQRYELHHKEEIQYGGEVFHVDNLCVLTPKRHISIHKDQ

*MIX regions are marked in yellow. GxxY core motif is underlined.*
