# Supplementary figures and images for: The Antibacterial and Anti-Eukaryotic Type VI Secretion System MIX-Effector Repertoire in Vibrionaceae
Source: Mar Drugs. 2018 Nov 4;16(11):433. doi: 10.3390/md16110433 (PMC6267618; doi:10.3390/md16110433)

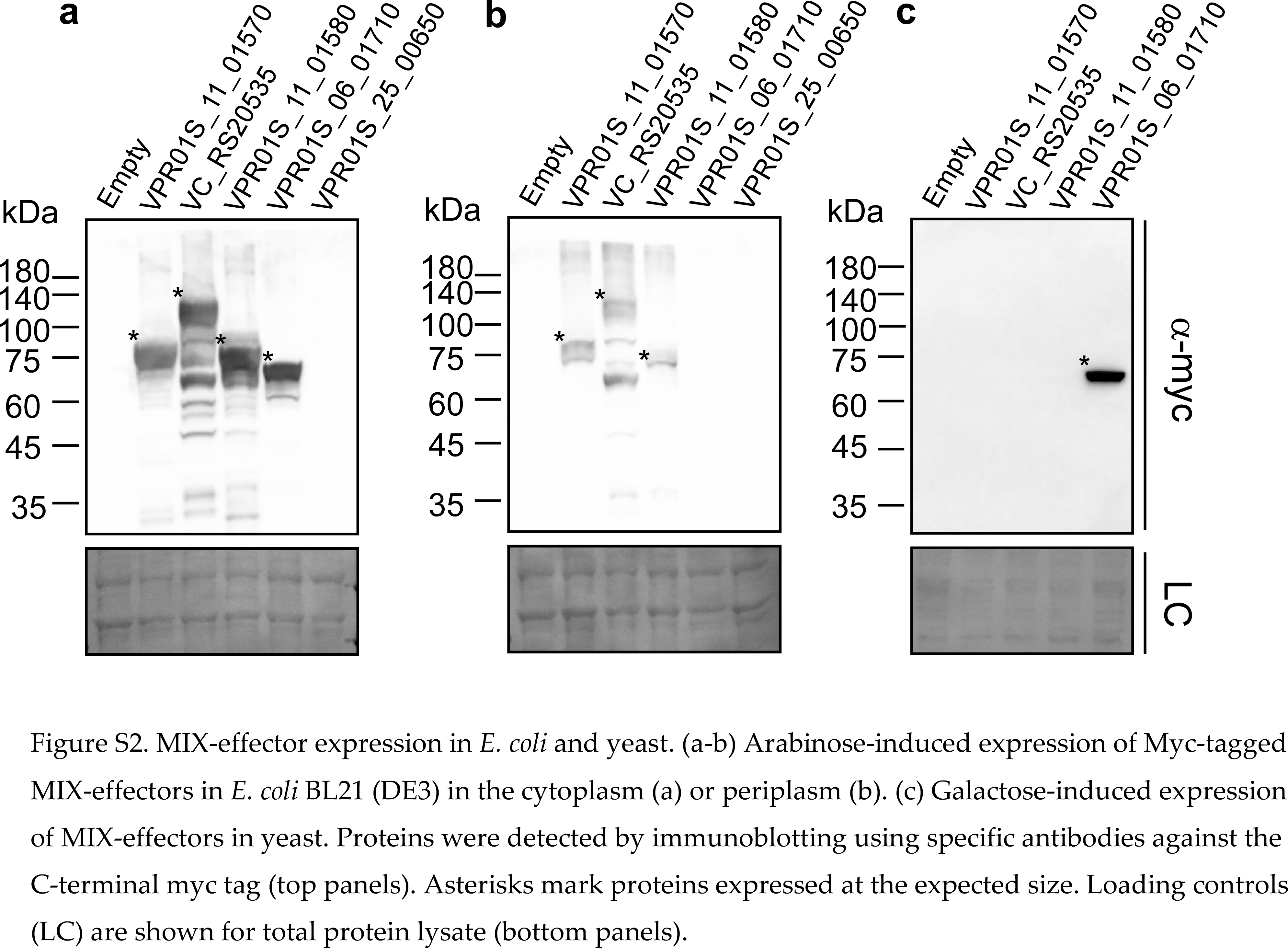

Supplement: Supplementary file 1 [file marinedrugs-16-00433-s001.zip › Figure S2_rev.tif]
